# Next-generation digital polymerase chain reaction assay for *BCR::ABL1* in chronic myeloid leukemia: comprehensive validation for standardized measurable residual disease detection

**DOI:** 10.1007/s44313-026-00188-5

**Published:** 2026-10-16

**Authors:** Yong-Hwi Kim, Min-Sik Jang, Shin-Hyun Kim, In-Young Roh, Bong-Suk Kim, Kyung-Mi Kee, Soo-Hyun Kim, Dong-Wook Kim, Jae-Hoon Kim

**Affiliations:** 1 BioTNS Co., Ltd., Daejeon, 34183 Republic of Korea; 2 Leukemia Omics Research Institute, Eulji University, Uijeongbu, 11759 Republic of Korea; 3 Department of Hematology, Hematology Center, Eulji Medical Center, Eulji University, Uijeongbu, 11759 Republic of Korea

**Keywords:** Analytical validation, Clinical validation, Digital PCR, *BCR::ABL1*, Chronic myeloid leukemia, Measurable residual disease

## Abstract

**Purpose:**

The sensitivity of real-time quantitative reverse-transcription polymerase chain reaction (RT-qPCR) for detecting measurable residual disease (MRD) in patients with chronic myeloid leukemia (CML) who have achieved a deep molecular response is limited. We validated a digital PCR (dPCR) assay (LQ Check BCR::ABL1 Major %IS Kit) for MRD quantification.

**Methods:**

Validation was performed according to CLSI guidelines EP17-A2, EP06, EP05-A3, and EP07 using *BCR::ABL1*-positive and -negative materials and the World Health Organization (WHO) 1st International Standard. A nonparametric analysis of the limit of blank was used to establish a positivity cutoff of ≥ 2 droplets. The limit of detection (LoD) was verified using a detection criterion of ≥ 95%, and the limit of quantitation (LoQ) was defined as the lowest level meeting ≥ 95% detection and a study-defined %CV of ≤ 76%. Clinical validation included 72 CML specimens for molecular response (MR) category comparisons and 67 specimens for paired quantitative comparisons.

**Results:**

The LoD and LoQ were both 0.0025% on the International Scale (IS) with a detection rate of 95.8%. The assay showed linearity over 5 logs (R^2^ = 0.9916–0.9972), within-run %CV ≤ 1.52%, cross-condition %CV ≤ 2.76%, and WHO-traceable accuracy. In 67 paired specimens, the correlation coefficient was r = 0.9740, with a Passing–Bablok slope of 1.0189, intercept of −0.2552, and Bland–Altman mean bias of 0.2266. Among the 72 specimens, dPCR classified 20 as having a deeper MR than RT-qPCR. Reproducibility was characterized by r = 0.99 and a mean bias of 0.0967. Whole blood was preferably processed within 48 h, with processing at 72 h considered acceptable only when *BCR* QC criteria were met.

**Conclusion:**

The LQ Check BCR::ABL1 Major %IS Kit enabled sensitive, precise, and reproducible *BCR::ABL1* quantification at MR4.6. The clinical significance of a deeper MR classification using dPCR, particularly for treatment-free remission, requires longitudinal validation.

**Supplementary Information:**

The online version contains supplementary material available at https://doi.org/10.1007/s44313-026-00188-5.

## Introduction

Chronic myeloid leukemia (CML) is a myeloproliferative neoplasm driven by the *BCR::ABL1* fusion gene arising from the reciprocal translocation t(9;22)(q34;q11) [1, 2]. This genetic rearrangement is present in > 90% of patients and produces three principal fusion transcript classes based on breakpoint location—Major (M‑*BCR*), Minor (m‑*BCR*), and Micro (μ‑*BCR*) [3]. The advent of tyrosine kinase inhibitors has dramatically improved prognosis, and achieving a deep molecular response (DMR) has enabled treatment-free remission (TFR) in selected patients [4]. Accordingly, sensitive and accurate quantification of *BCR::ABL1* transcripts, which serve as key molecular markers of measurable residual disease (MRD), is central to clinical decision-making, including decisions regarding treatment modification and potential discontinuation [4, 5].

Molecular response (MR) is reported on the International Scale (%IS), with standard thresholds of MR1 (10%IS), MR2 (1%IS), MR3 (0.1%IS), MR4 (0.01%IS), and MR4.5 (0.0032%IS) [6, 7]. In real-world practice, reliable MRD measurements in the DMR range are essential for safe TFR attempts [8]. However, the current standard, real-time quantitative reverse-transcription polymerase chain reaction (RT‑qPCR), relies on standard curves and is subject to inter-laboratory and inter-operator variability [9]. Variability is particularly high at or beyond MR4.5, potentially compromising measurement reliability and increasing the risk of false-negative results [10, 11].

Droplet digital PCR (dPCR) has emerged as a promising alternative, enabling absolute quantification by partitioning reactions into numerous microdroplets and estimating target copy numbers using Poisson statistics. It also demonstrates robustness to variations in PCR efficiency [12, 13]. DPCR can detect low levels of *BCR::ABL1* transcripts in the MR4.5–MR5 range [12, 14]. Moreover, dPCR results can be converted to %IS through calibration against World Health Organization (WHO) international standards [11].

Accordingly, this study aimed to evaluate the analytical and clinical validity of a newly designed dPCR assay that simultaneously quantifies the major CML transcript variants, e13a2 and e14a2, and the *BCR* reference gene, in a single reaction. Specifically, we sought to validate key analytical performance characteristics—sensitivity, accuracy, precision, and specificity—according to international guidelines and compare the assay’s performance with that of conventional RT‑qPCR using clinical specimens to assess its clinical applicability.

## Materials and methods

### Patient samples

Overall, 104 patients with CML were included in this study. Clinical performance and reproducibility were assessed using anonymized total RNA from 79 and 10 patients, respectively, obtained retrospectively from GC Labs (IRB No. GCL-2024–2024-06), and Uijeongbu Eulji Medical Center (IRB No. UEMC 2022–01–019) biobanks. Among the 79 clinical performance specimens, seven failed the initial RNA quality control because the OD_260_/OD_230_ ratio was < 1.2, leaving 72 specimens for categorical clinical comparison. Of these, 5 yielded negative dPCR results according to the predefined reporting criterion (≤ 1 positive droplet, below the limit of blank [LoB]) and were included in categorical comparison but not in paired log-transformed quantitative analyses, leaving 67 specimens for Passing–Bablok regression and Bland–Altman analysis. Peripheral blood samples from 15 adult patients with CML were collected at the Uijeongbu Eulji Medical Center, anonymized, and used for analysis (IRB No. UEMC 2025–04–011).

### Cell line samples

Five cell lines (K-562, KCL-22, HL-60, AR230-r, and SUP-B15) and *BCR-ABL1* WHO standard panels (08/192, 08/194, 08/196, and 08/198) were used. K-562 (ATCC CCL-243™), AR230-r (e1a2-transcript CML, ATCC CRL-3346), SUP-B15 (ALL, ATCC CRL-1929), and HL-60 (APL, ATCC CCL-240™) were purchased from ATCC; KCL-22 (JCRB1317) was purchased from the Japanese Collection of Research Bioresources Cell Bank; and WHO standard panels were purchased from NIBSC. Cells were cultured under supplier-recommended conditions [15] and maintained at > 90% viability.

### Total RNA extraction and cDNA synthesis

Total RNA from blood specimens and cell lines was extracted using the High Pure RNA Isolation Kit (F. Hoffmann-La Roche Ltd., Mannheim, Germany) according to the manufacturer’s instructions and quantified and quality-checked (OD_260_/OD_280_ and OD_260_/OD_230_ ratios) using NanoDrop One/OneC (Thermo Fisher Scientific Inc., Waltham, MA, USA). cDNA was synthesized using the Transcriptor First Strand cDNA Synthesis Kit (F. Hoffmann-La Roche Ltd., Mannheim, Germany) following the manufacturer’s instructions.

### Whole-blood specimen stability

Whole blood from 15 adult patients with CML was collected in 10-mL EDTA tubes and stored at 2–8 °C. Cell lysates were prepared at approximately 24, 48, 72, and 96 h and assessed for *BCR* reference gene expression using LQ Suite dPCR. At each time point, 1,000 ng of total RNA per specimen was tested in 10 replicates. Specimens were considered stable when *BCR* was ≥ 2,000 copies/μL at all time points; lysates were stored at ≤ − 70 °C until analysis.

### LoB

LoB was determined using total RNA from *BCR::ABL1*-negative HL-60 cells, and two lots of the LQ Check BCR::ABL1 Major %IS Kit (BioTNS Co., Ltd., Daejeon, Republic of Korea) were tested in 60 replicates. According to CLSI EP17-A2 [16], a nonparametric approach designated the 95th percentile (mean of the 57th and 58th ordered measurements) as the LoB.

### Limit of Detection (LoD)

LoD was established per CLSI EP17-A2 [16] using e13a2/e14a2 panels at six levels (0.0010%IS–0.0032%IS, MR4.5–MR5.0; 24 replicates/level across 2 lots; 576 data points). Probit analysis via the GLM function in IBM SPSS Statistics v25 (IBM Corp., Armonk, NY, USA) and a nonparametric criterion (≥ 95% hit rate) were applied [6].

### Limit of Quantitation (LoQ)

The LoQ was derived from the LoD dataset by calculating %IS-based coefficient of variation (%CV) for e13a2 and e14a2 at concentrations ≥ LoD. The LoQ was defined as the lowest level meeting %CV ≤ 76% [17]. This threshold was adopted as a study-specific acceptance criterion based on the FDA-cleared QXDx assay precedent [17] rather than as a universal CLSI-recommended cutoff and was applied specifically to the assay evaluated in this study.

### Linearity

Linearity was evaluated using CLSI EP06 [18]. Positive RNA from KCL-22 (e13a2) and K-562 (e14a2) was quantified in %IS using the Real-Q BCR-ABL Quantification Kit (BioSewoom Inc., Seoul, Republic of Korea) and serially diluted with HL-60 RNA to six levels (10.4301%IS [MR0.97] to 0.0006%IS [MR5.26]). Each level was measured in triplicate across two lots of the LQ Check BCR::ABL1 Major %IS Kit. Linear regression evaluated log_10_-transformed expected versus observed values, with acceptance criteria of slope 0.83–1.20 and R^2^ 0.97 [19].

### Analytical precision

Precision was evaluated per CLSI EP05-A3 [20] and Korean Ministry of Food and Drug Safety guidelines [21] using K-562 (*BCR::ABL1*-positive) spiked into HL-60 (*BCR::ABL1*-negative) at 1%IS (MR2), 0.1%IS (MR3), and 0.01%IS (MR4). Repeatability was assessed under single conditions (1 lot, 1 instrument, and 1 operator; 2 runs/day, 3 replicates/run for 20 days). Reproducibility was tested across three lots, sites, instruments, and operators (2 runs/day, 3 replicates/run for 5 days).

### Analytical specificity

Interference was assessed per CLSI EP07 [22] by spiking potential interferents into low-positive e14a2 RNA (0.1%IS, MR3) and a negative whole-blood matrix. Cholesterol (400 mg/dL), bilirubin (40 mg/dL), hemoglobin (1000 mg/dL), triglyceride (1500 mg/dL), albumin (10 g/dL), heparin (330 units/dL), guanidinium-containing lysis buffer (1%), EDTA (3.4 μmol/L), EtOH (0.1%), and genomic DNA (50 ng/RT) were tested in triplicate.

### Analytical accuracy

Accuracy was evaluated per CLSI EP06 [18] using the WHO 1 st International Standard panel (NIBSC code: 09/138; 4 levels, 0.0195%IS–16.3129%IS) tested in triplicate across three lots. Acceptance criteria were slope 0.83–1.20 and R^2^ ≥ 0.97 [19].

### Clinical validation

The clinical performance was assessed using 79 retrospectively collected specimens. Seven samples failed the predefined RNA quality control criterion (OD_260_/OD_230_ ratio < 1.2), leaving 72 samples for categorical comparison between LQ Suite dPCR and real-time quantitative reverse-transcription polymerase chain reaction (RT-qPCR). Five specimens showed low-level positivity by RT-qPCR (MR3.92–4.47) but yielded negative dPCR results according to the predefined reporting criterion (≤ 1 positive droplet, below the LoB). These results were treated as valid categorical negative results and were included in the MR-category agreement analyses. However, because negative dPCR results could not be log_10_-transformed, these five specimens were excluded from the paired quantitative analyses. Therefore, Passing–Bablok regression and Bland–Altman analyses were performed using the 67 specimens with quantitative results from both methods. Categorical comparisons used Cohen’s kappa coefficient for MR-category agreement. For the 67 quantifiable specimens, %IS comparison used Passing–Bablok regression and Bland–Altman analysis with the following criteria: r ≥ 0.95 [23], evaluation of proportional and systematic biases via 95% confidence intervals (CIs) for slope and intercept (including 1 and 0, respectively) [24], and overall clinical agreement within a mean bias of ±0.5 log_10_ [14]. Reproducibility assessed 10 specimens (MR, 0.04–3.93) across three sites, instruments, and operators versus RT-qPCR.

### RT-qPCR assays

RT-qPCR was performed as the comparator using the Real-Q BCR-ABL Quantification Kit (BioSewoom Inc., Seoul, Republic of Korea). cDNA from 1 µg total RNA was analyzed on a QuantStudio™ 5 system (Thermo Fisher Scientific Inc., Waltham, MA, USA) per the kit protocol. The *BCR::ABL1*/*ABL1* ratio was converted to %IS and MR using a validated laboratory conversion factor.

### LQ suite dPCR assays

*BCR::ABL1* was quantified using the LQ Check BCR::ABL1 Major %IS Kit in the LQ Suite dPCR system (BioTNS Co., Ltd., Daejeon, Republic of Korea). Reaction mixtures were partitioned using the LQ Suite Droplet Generator, transferred to 96-well plates, and amplified on a SimpliAmp™ Thermal Cycler (Thermo Fisher Scientific Inc., Waltham, MA, USA) at 95 °C for 10 min, followed by 45 cycles of 94 °C for 30 s and 58 °C for 60 s (2.5 °C/s), and then 98 °C for 10 min. Droplet fluorescence was read using an LQ Suite Droplet Analyzer, and %IS/MR was calculated using NanoFlex software version 1.2.1 (BioTNS Co., Ltd., Daejeon, Republic of Korea).

### Statistical analyses

*BCR::ABL1* stability was assessed at 24, 48, 72, and 96 h using repeated-measures analysis of variance (ANOVA). Normality was assessed using the Shapiro–Wilk test with Greenhouse–Geisser correction for violated sphericity and Bonferroni-adjusted pairwise comparisons. For analytical performance, linearity was evaluated using polynomial and linear regression analyses, and analytical precision (repeatability and reproducibility) was expressed as %CV. Clinical comparisons used Cohen’s kappa coefficient for MR-category agreement, Passing–Bablok regression for quantitative correlation, and Bland–Altman analysis for mean bias and 95% limits of agreement.

Analyses used MedCalc v23.3.5 (MedCalc Software Ltd., Ostend, Belgium), IBM SPSS Statistics v25 (IBM Corp., Armonk, NY, USA), and Microsoft Excel® (Microsoft Corp., Redmond, WA, USA). *P* < 0.05 was considered statistically significant.

## Results

### Expression of reference gene transcript

To ensure the reliability of the quantitative values obtained from the method used in this study, the minimum required expression level for the *BCR* reference gene was recalculated based on the minimum cellular *ABL1* expression threshold. Consequently, the minimum required expression level for the *BCR* reference gene was ≥ 2,000 copies/µL (40,529 copies/rxn), in accordance with established molecular monitoring benchmarks [25–27] (Table 1).

**Table 1:** Criteria for validity assessment of the minimum concentration of the *BCR* reference gene

Terminology	*BCR::ABL1*%IS	Minimum *ABL1* control transcript numbers^a^	Minimum *BCR* control transcript numbers^b^
Equivalent to CCyR	≤ 1%IS	10,000	12,665
MMR	≤ 0.1%IS	10,000	12,665
4 log reduction from the IRIS standardized baseline (MR4)	≤ 0.01%IS	10,000	12,665
4.5 log reduction from the IRIS standardized baseline (MR4.5)	≤ 0.0032%IS	32,000	40,529
5 log reduction from the IRIS standardized baseline (MR5)	≤ 0.001%IS	100,000	126,653

^a^[25]

^b^The intracellular transcript expression level of the *BCR* gene was calculated based on the WHO primary reference standard values determined by Cross et al. [26] and the minimum threshold of the *ABL1* gene determined by Apperley et al. [25]. Iland et al. [27] noted that > 30,000 *BCR* transcripts were adequate for valid *BCR‑ABL*/*BCR*% results, which is a general RNA quality benchmark that is not tied to any specific MR level. Our minimum *BCR* control level (≥ 2,000 copies/μL, 40,529 copies/rxn) exceeds this threshold. CCyR, complete cytogenetic remission; %IS, % International Scale; MMR, major molecular response; MR, molecular response; WHO, World Health Organization

### Whole-blood specimen stability

**Fig. 1:**
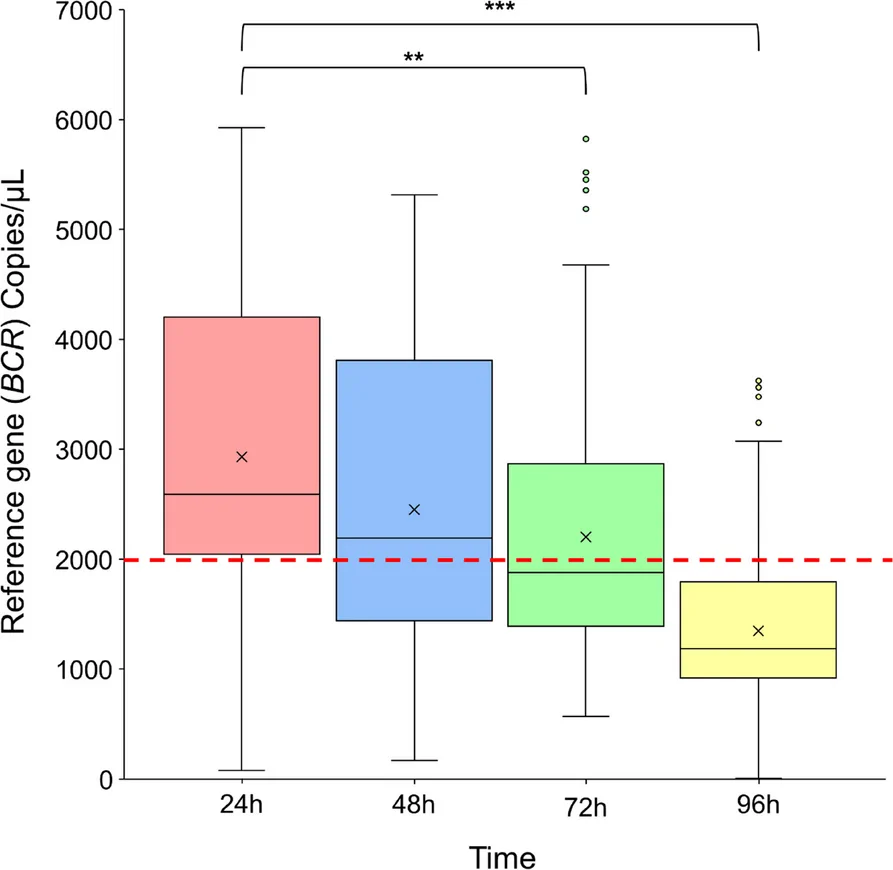
Assessment of *BCR* reference gene transcript stability in whole-blood samples from 15 patients with CML at 24, 48, 72, and 96 h after collection. The box-and-whisker plots represent the distribution of copy numbers (copies/μL) at each time point. The horizontal line within the box indicates the median, and the “ × ” mark represents the mean. The top and bottom boundaries of the box correspond to the 75th and 25th percentiles (interquartile range), respectively, whereas the whiskers extend to the maximum and minimum values excluding outliers. Outliers are plotted as individual points. The red dashed line indicates the minimum validity threshold of 2,000 copies/μL. Statistical significance was determined using repeated measures analysis of variance with Bonferroni correction. Asterisks indicate statistically significant differences compared with the 24 h baseline or between indicated time points (^**^P < 0.01, ^***^P < 0.001). CML, chronic myeloid leukemia

Whole-blood samples from 15 adult patients with CML were stored at 2–8 °C, and *BCR* control-gene copy numbers (copies/μL) were measured at 24, 48, 72, and 96 h using the LQ Suite dPCR system, with 10 replicates per patient (*n* = 150 per time point, *N* = 600 overall). At each time point, the mean copy numbers and standard deviations were as follows: 24 h, 2929.7 ± 1508.9; 48 h, 2449.6 ± 1375.8; 72 h, 2201.4 ± 1168.7; and 96 h, 1346.6 ± 831.5 copies/μL. The median values were as follows: 24 h, 2589.05; 48 h, 2190.88; 72 h, 1876.66; and 96 h, 1186.13, with corresponding interquartile ranges of 2120.5, 2284.9, 1461.0, and 847.1, respectively. Repeated-measures ANOVA showed significant group differences (*P* < 0.001, Greenhouse–Geisser correction). Bonferroni-adjusted post hoc analysis confirmed that copy numbers at 96 h were significantly lower than those at earlier time points (*P* < 0.001); the decrease was also significant between 24 and 72 h (*P* = 0.001), but the differences between adjacent early time points were not significant. The proportion of samples meeting the assay validity threshold (≥ 2,000 copies/μL) declined over time, and at 96 h, only a minority remained valid (Fig. 1). In summary, *BCR* reference gene copy number declined significantly between 24 and 72 h (*P* = 0.001). RNA extraction within 48 h was therefore preferred for routine testing; 72 h should be considered an exceptional upper limit under 2–8 °C storage, and results should be reported only when the *BCR* validity threshold (≥ 2,000 copies/μL) is met.

### LoB

The LoB for the LQ Check BCR::ABL1 Major %IS Kit was determined using total RNA extracted from the HL-60 cell line. As the measurement values were not normally distributed, a CLSI EP17‑A2-based nonparametric approach was applied to 60 replicates ordered in ascending rank; the 95th percentile (the 57.5th position) corresponded to the 57–58th observations, yielding a *BCR::ABL1* LoB of one positive droplet. To ensure specificity based on this nonparametric LoB analysis, the positivity cut‑off was set at ≥ 2 positive droplets (≈0.14 copies/μL) (Supplemental Table 1). Setting a stringent cutoff is paramount in the context of CML management, as it minimizes the risk of false-positive results that can incorrectly influence crucial decisions.

### LoD

**Fig. 2:**
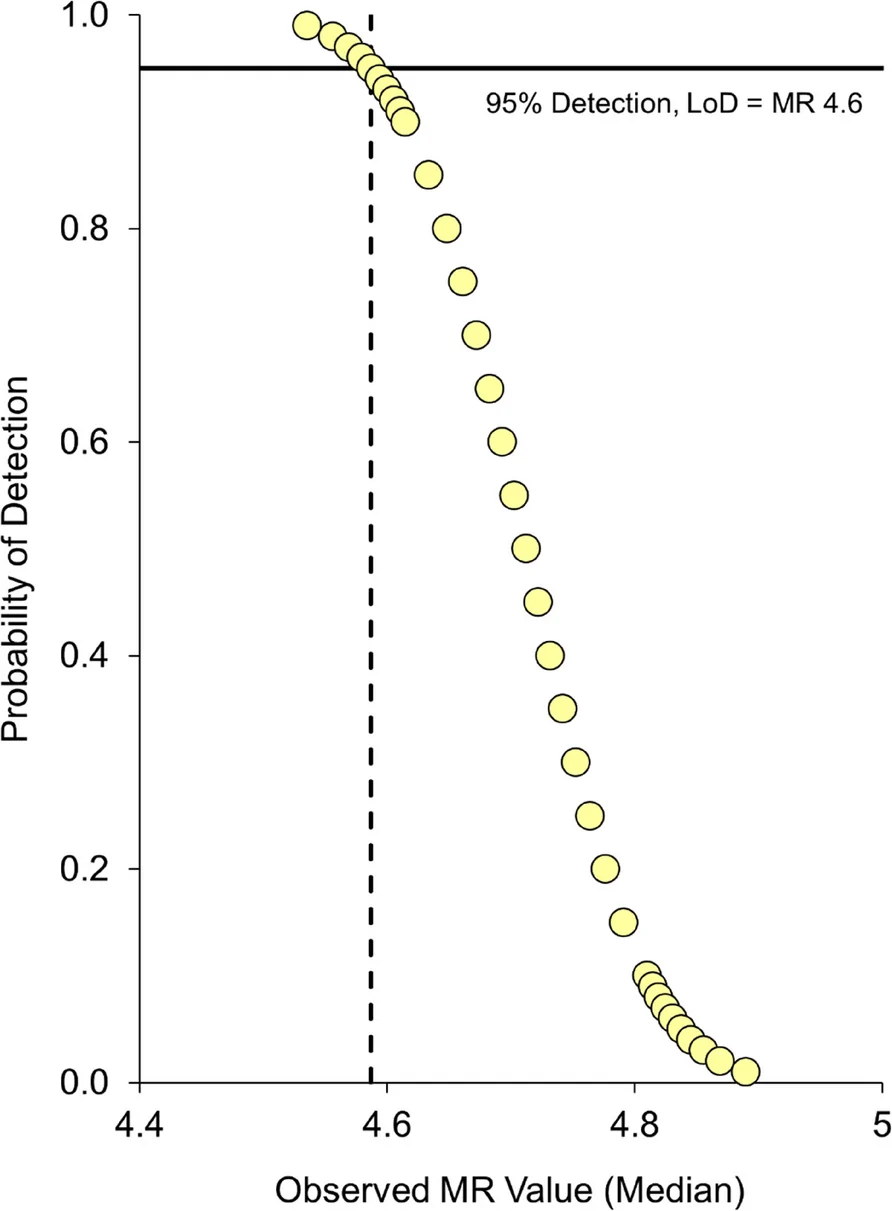
Probit-based estimation of the LoD. Six serial dilution levels of cell line–derived panels (K‑562 and KCL‑22 spiked into HL‑60) were tested in two reagent lots for e13a2 and e14a2, yielding 576 measurements. For each dilution level, the median MR value from 96 replicate reactions (24 replicates for each transcript type in each of two lots) is plotted on the *x*-axis, and the corresponding proportion of positive results is plotted on the *y*-axis. The probit model indicates that the detection probability reaches 95% at MR4.60 (0.0025%IS), and the point estimate at this probability level is MR4.59, marked by the intersection of the vertical dashed line and the horizontal 95% probability line. The same model predicts detection probabilities of approximately 85% at MR4.63, 75% at MR4.66, 50% at MR4.71, and 25% at MR4.76. Nonparametric verification at MR4.60 confirmed a ≥ 95% detection rate for each transcript type (Table 2). %IS, % International Scale; LoD, limit of detection, MR, molecular response

The LoD was evaluated for fusion genotypes (e13a2 and e14a2) across two lots using serial dilution panels spanning 0.0032%IS (MR4.5) to 0.0010%IS (MR5.0) prepared by spiking K‑562 and KCL‑22 into HL‑60, with probit regression applied to 576 valid measurements (24 replicates per level) to estimate the concentration achieving ≥ 95% detection (Fig. 2). Both genotypes achieved a 95.8% hit rate at 0.0025%IS (MR4.6), with transcript-specific LoD values of MR4.5990 (e13a2) and MR4.5770 (e14a2). Additional model‑estimated probabilities were 85% at MR4.633 (0.0023%IS), 75% at MR4.659 (0.0022%IS), 50% at MR4.71 (0.0019%IS), and 25% at MR4.76 (0.0017%IS). Nonparametric verification confirmed 95.8333% detection at MR4.60 for both breakpoints (Table 2).

**Table 2:** LoD results for each *BCR::ABL1* transcript type (e13a2 and e14a2) based on cell line–derived serial dilution panels (K‑562 and KCL‑22 spiked into HL‑60)

Transcript identity	Total measurements	Positive results	Negative results	Detected, %	LoD (MR)	LoD (%IS)
e13a2	48	46	2	95.8333	4.60	0.0025
e14a2	48	46	2	95.8333	4.60	0.0025
e13a2 + e14a2	96	92	4	95.8333	4.60	0.0025

LoD was estimated using Probit analysis (Fig. 2) and nonparametrically validated, confirming that > 95% of all low-level samples targeting the expected LoD of MR4.6 (≥ 0.0025%IS) were detected. The MR measurements served as the final acceptance criteria. %IS, % International Scale; MR, molecular response; LoD, limit of detection

### LoQ

The LoQ was evaluated across six concentrations from 0.0032%IS (MR4.5) to 0.0010%IS (MR5.0) using cell line–derived serial dilution panels (K‑562 and KCL‑22 spiked into HL‑60) for two fusion transcripts (e13a2 and e14a2) and two production lots (lots 1 and 2) and was defined as the lowest level meeting both a > 95% positive detection rate and a %CV ≤ 76%. At 0.0025%IS (MR4.6), the %CVs were 42.21% (lot 1) and 41.60% (lot 2) for e13a2 and 37.83% (lot 1) and 52.06% (lot 2) for e14a2, confirming that both transcripts met the %CV ≤ 76% criterion at 0.0025%IS (MR4.6) (Table 3, Supplemental Table 2).

**Table 3:** LoQ results for each *BCR::ABL1* transcript type (e13a2, e14a2) based on cell line–derived serial dilution panels (K‑562 and KCL‑22 spiked into HL‑60)

Lot	Transcript identity	Total specimens	Positive specimens	Positive detection rate (%)	CV (%)		LoQ		Results
					%IS	MR	%IS	MR	
1	e13a2	24	23	95.83	42.21	5.13	0.0025	4.60	Pass
1	e14a2	24	23	95.83	37.83	4.17	0.0025	4.60	Pass
2	e13a2	24	23	95.83	41.60	4.37	0.0025	4.60	Pass
2	e14a2	24	23	95.83	52.06	4.30	0.0025	4.60	Pass

*CV* coefficient of variation, *%IS* % International Scale, *LoQ* limit of quantitation, *MR* molecular response

### Linearity

**Fig. 3:**
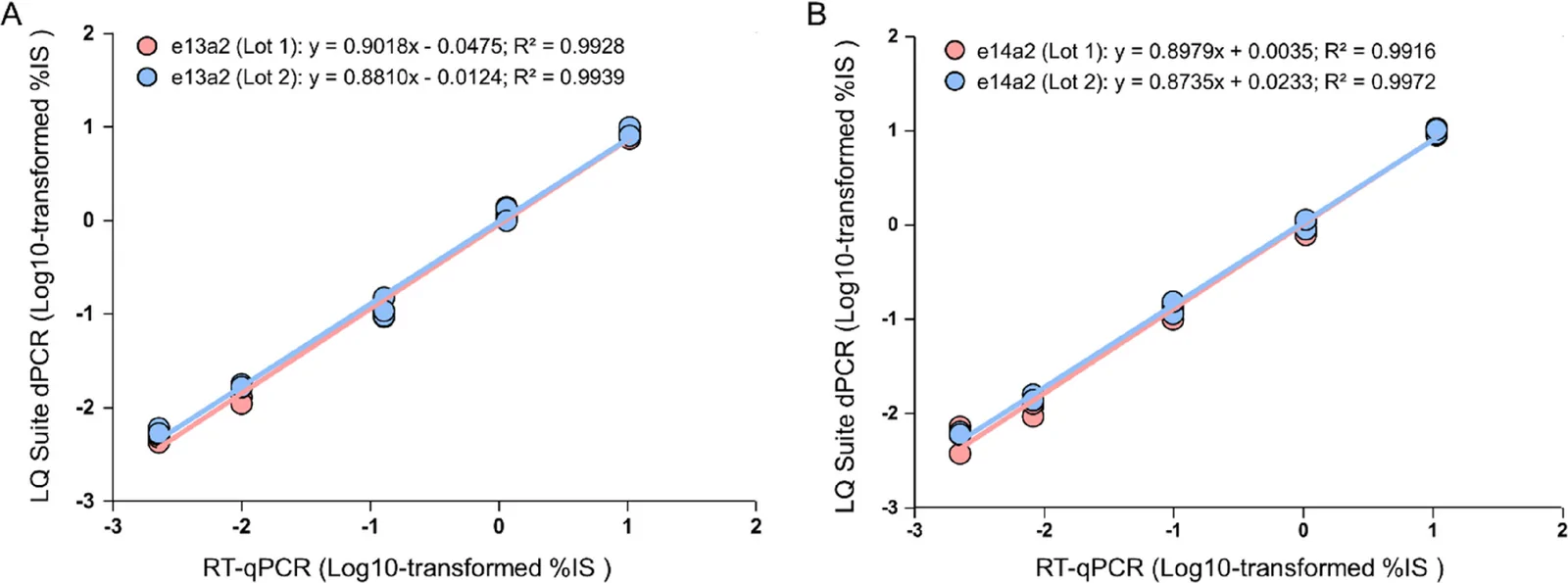
Evaluation of linearity for *BCR::ABL1* major fusion transcripts. Linearity was assessed by measuring log_10_-transformed %IS values across a serial dilution range spanning approximately 5 orders of magnitude. **A** Linearity for the e13a2 transcript was evaluated using KCL-22 cell line dilutions (lot 1: red circles, lot 2: blue circles). **B** Linearity for the e14a2 transcript was evaluated using K-562 cell line dilutions. The *x*-axis represents the expected log_10_-transformed %IS values derived from RT-qPCR, and *y*-axis represents the observed log_10_-transformed %IS values measured using the LQ Suite dPCR system. Solid lines indicate the linear regression fit for each lot. Regression equations and coefficients of determination (R^2^) are displayed within the plot for each lot, demonstrating strong linear correlation (R^2^ > 0.99) across the tested range (e13a2: 10.4301%IS to 0.0006%IS, e14a2: 10.6813%IS to 0.0010%IS). dPCR, digital PCR; %IS, % International Scale; RT-qPCR, real-time quantitative reverse-transcription polymerase chain reaction

Linearity was evaluated separately for e13a2 and e14a2 using cell line–derived serial dilution panels (K‑562 and KCL‑22 spiked into HL‑60), spanning 10.4301%IS (MR0.9817) to 0.0006%IS (MR5.2596) for e13a2 and 10.6813%IS (MR0.9714) to 0.0010%IS (MR4.9792) for e14a2. Across two production lots (lots 1 and 2), linear regression on log_10_‑transformed expected versus observed values yielded lot 1 y = 0.9018x − 0.0475 with R^2^ = 0.9928 and lot 2 y = 0.881x − 0.0124 with R^2^ = 0.9939 for e13a2 and lot 1 y = 0.8979x + 0.0035 with R^2^ = 0.9916 and lot 2 y = 0.8735x + 0.0233 with R^2^ = 0.9972 for e14a2, indicating strong correlation across the entire range (Fig. 3). The mean %IS and MR values were continuously measured from high (10%IS) to low (0.0006%IS) concentrations across both transcripts and lots, and overall linearity R^2^ values fell between 0.9916 and 0.9972, confirming robust linearity over the stated dilution ranges (Supplemental Tables 3 and 4).

### Analytical precision

Analytical precision was assessed using cell lines at high, medium, and low concentrations across run, lot, site, instrument, and operator, showing within‑run %CVs ≤ 1.52% at all levels, specifically 1.52% (high), 1.43% (medium), and 1.33% (low), indicating low variability under identical conditions (Table 4). For reproducibility, %CVs across lots, sites, instruments, and operators ranged from 0.20% to 2.76%, with particularly low between‑instrument and between‑operator %CVs of 0.20% and 0.31%, respectively, confirming minimal variation attributable to manufacturing, testing environment, instruments, or operators (Table 4).

**Table 4:** Analytical precision of the LQ Check BCR::ABL1 Major %IS Kit on the LQ Suite dPCR System summarizing repeatability and reproducibility according to source of variation with SD and %CV for high, middle, and low positive samples

Evaluation parameter	Source of variation	Sample	Mean	Within-run		Between-run		Between-day		Within-laboratory		Between-lot		Between-site		Between-instrument		Between-operator	
				SD	%CV	SD	%CV	SD	%CV	SD	%CV	SD	%CV	SD	%CV	SD	%CV	SD	%CV
Repeatability	-	High^a^	1.94	0.03	1.52	0.05	2.62	0.04	2.16	0.07	3.64	-	-	-	-	-	-	-	-
		Middle^b^	2.95	0.04	1.43	0.06	1.91	0.05	1.52	0.09	2.97	-	-	-	-	-	-	-	-
		Low^c^	3.97	0.05	1.33	0.06	1.51	0.05	1.23	0.09	2.34	-	-	-	-	-	-	-	-
Reproducibility	Between-lot	High^a^	1.98	0.04	2.25	0.02	1.02	0.03	1.52	0.08	4.01	0.05	2.76	-	-	-	-	-	-
		Middle^b^	3.00	0.06	2.01	0.03	1.08	0.04	1.33	0.10	3.20	0.05	1.81	-	-	-	-	-	-
		Low^c^	4.00	0.07	1.81	0.04	0.89	0.04	1.09	0.12	2.90	0.07	1.77	-	-	-	-	-	-
	Between-site	High^a^	1.94	0.04	1.85	0.03	1.29	0.04	2.18	0.06	3.15	-	-	0.01	0.31	-	-	-	-
		Middle^b^	2.93	0.05	1.63	0.03	0.88	0.05	1.70	0.08	2.62	-	-	0.02	0.76	-	-	-	-
		Low^c^	3.95	0.07	1.66	0.04	0.96	0.06	1.45	0.10	2.45	-	-	0.02	0.49	-	-	-	-
	Between-instrument	High^a^	1.94	0.03	1.74	0.02	1.25	0.06	3.11	0.07	3.78	-	-	-	-	0.00	0.20	-	-
		Middle^b^	2.93	0.04	1.34	0.04	1.41	0.04	1.30	0.07	2.36	-	-	-	-	0.01	0.32	-	-
		Low^c^	3.98	0.05	1.14	0.07	1.76	0.07	1.86	0.11	2.83	-	-	-	-	0.01	0.36	-	-
	Between-operator	High^a^	2.02	0.05	2.32	0.03	1.44	0.03	1.38	0.06	3.08	-	-	-	-	-	-	0.01	0.31
		Middle^b^	3.04	0.07	2.36	0.04	1.36	0.04	1.29	0.09	3.05	-	-	-	-	-	-	0.02	0.50
		Low^c^	4.05	0.07	1.65	0.03	0.68	0.03	0.76	0.08	2.04	-	-	-	-	-	-	0.03	0.63

^a^High positive specimen (MR2, 1%IS)

^b^Middle positive specimen (MR3, 0.1%IS)

^c^Low positive specimen (MR4, 0.01%IS)

*CV* coefficient of variation, *%IS* % International Scale, *MR* molecular response, *SD* standard deviation

### Analytical specificity

Analytical specificity was evaluated using interference and cross-reactivity testing. Endogenous interferents (cholesterol, bilirubin, hemoglobin, and triglycerides), exogenous substances (heparin, guanidinium-containing lysis buffer, EDTA, and EtOH), medications, and genomic DNA showed no significant effects on %IS or MR in positive or negative samples (*P* > 0.05). Even when the interferents were combined, the mean, SD, bias, and %CV met the acceptance criteria (Table 5). Cross-reactivity testing of hematological cancer cell lines, normal human blood, and related gene RNA showed no nonspecific *BCR::ABL1* detection; internal controls and the *BCR* reference gene were consistently detected, confirming the assay validity (Table 6).

**Table 5:** Analytical specificity evaluation of the LQ Check BCR::ABL1 Major %IS Kit on the LQ Suite dPCR System summarizing interference study results for various endogenous and exogenous substances, with SD and %CV

Materials	Samples	%IS				MR level			
		Mean	SD	Bias	%CV	Mean	SD	Bias	%CV
None	Positive sample (0.1%IS, MR3)	0.10	0.01	0.00	6.09	2.99	0.03	–0.01	0.89
	Negative sample	0.00	0.00	0.00	N/A	N/A	N/A	N/A	N/A
Cholesterol	Positive sample (0.1%IS, MR3)	0.11	0.01	0.01	10.53	2.96	0.04	–0.04	1.50
	Negative sample	0.00	0.00	0.00	N/A	N/A	N/A	N/A	N/A
Bilirubin	Positive sample (0.1%IS, MR3)	0.10	0.01	0.00	15.09	3.02	0.07	0.02	2.26
	Negative sample	0.00	0.00	0.00	N/A	N/A	N/A	N/A	N/A
Hemoglobin	Positive sample (0.1%IS, MR3)	0.11	0.01	0.01	10.34	2.94	0.05	–0.06	1.58
	Negative sample	0.00	0.00	0.00	N/A	N/A	N/A	N/A	N/A
Triglyceride	Positive sample (0.1%IS, MR3)	0.10	0.03	0.00	25.93	3.02	0.12	0.02	3.85
	Negative sample	0.00	0.00	0.00	N/A	N/A	N/A	N/A	N/A
Albumin	Positive sample (0.1%IS, MR3)	0.12	0.01	0.02	11.98	2.93	0.05	–0.07	1.73
	Negative sample	0.00	0.00	0.00	N/A	N/A	N/A	N/A	N/A
Heparin	Positive sample (0.1%IS, MR3)	0.12	0.00	0.02	3.21	2.92	0.01	–0.08	0.48
	Negative sample	0.00	0.00	0.00	N/A	N/A	N/A	N/A	N/A
Guanidinium-containing lysis buffer	Positive sample (0.1%IS, MR3)	0.11	0.02	0.01	17.85	2.95	0.08	–0.05	2.77
	Negative sample	0.00	0.00	0.00	N/A	N/A	N/A	N/A	N/A
EDTA	Positive sample (0.1%IS, MR3)	0.10	0.02	0.00	23.87	3.00	0.10	0.00	3.29
	Negative sample	0.00	0.00	0.00	N/A	N/A	N/A	N/A	N/A
EtOH	Positive sample (0.1%IS, MR3)	0.11	0.00	0.01	3.48	2.97	0.02	–0.03	0.51
	Negative sample	0.00	0.00	0.00	N/A	N/A	N/A	N/A	N/A
gDNA	Positive sample (0.1%IS, MR3)	0.14	0.01	0.04	5.45	2.87	0.02	–0.13	0.84
	Negative sample	0.00	0.00	0.00	N/A	N/A	N/A	N/A	N/A

*CV* coefficient of variation, *Gdna* genomic DNA, *%IS* % International Scale, *MR* molecular response, *SD* standard deviation

**Table 6:** Analytical specificity evaluation of the LQ Check BCR::ABL1 Major %IS Kit on the LQ Suite dPCR System summarizing cross-reactivity study results for various cell lines, with SD and %CV

Samples	RNA input (ng)	Replicates	Valid droplets	*BCR::ABL1*		*BCR*		%IS	MR
				Positive droplets	Concentration (copies/uL)	Positive droplets	Concentration (copies/uL)		
AR230-r (ATCC CRL-3346)	100 ng	1	17,652	0	0.00	17,428	10,999.90	N/A	N/A
		2	17,775	0	0.00	17,540	10,896.63	N/A	N/A
		3	17,555	0	0.00	17,342	11,112.85	N/A	N/A
SUP-B15 (ATCC CRL-1929)	100 ng	1	20,379	0	0.00	19,936	9,644.06	N/A	N/A
		2	17,723	0	0.00	17,315	9,499.63	N/A	N/A
		3	18,012	0	0.00	17,572	9,350.17	N/A	N/A
HL 60 cell (ATCC CCL-240)	100 ng	1	17,396	0	0.00	17,245	11,956.46	N/A	N/A
		2	19,813	0	0.00	19,481	10,299.64	N/A	N/A
		3	16,826	0	0.00	16,647	11,444.07	N/A	N/A
Single Donor Human Whole Blood	100 ng	1	16,782	0	0.00	16,612	11,567.41	N/A	N/A
		2	18,144	0	0.00	17,820	10,139.42	N/A	N/A
		3	17,763	0	0.00	17,489	10,508.17	N/A	N/A

*CV* coefficient of variation, *dPCR* digital PCR, *gDNA %IS* % International Scale, *MR* molecular response, *N/A* not available, *SD* standard deviation

### Analytical accuracy

**Fig. 4:**
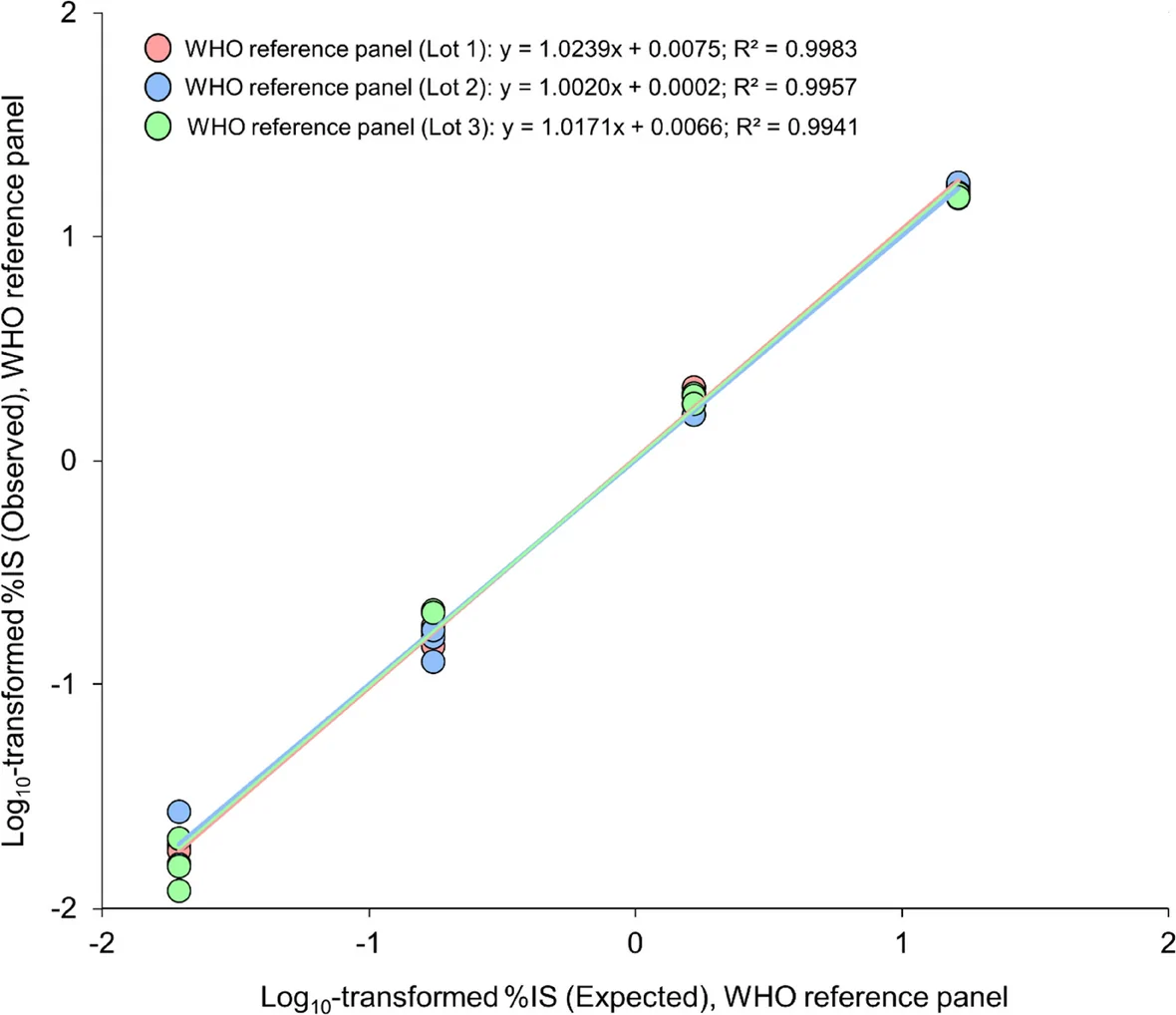
This study assessed the traceability of tertiary reference materials (kit calibrators) to the 1RM across three lots from a single manufacturing batch of the kit. Empirical MR values for the WHO primary (1RM) materials generated using the kit (*y*-axis) are plotted against the MR values published in the World Health Organization primary reference panel’s instructions for use (*x*-axis). The three lots from one batch are represented by red (lot 1), blue (lot 2), and green (lot 3) data points evaluated with three independent lots (different from linearity lots). %IS, % International Scale; MR, molecular response; WHO, World Health Organization

Analytical accuracy was evaluated using the WHO reference standard by regressing log₁₀‑transformed *BCR::ABL1*/*ABL1* (%IS) expected values (RT‑qPCR) against measured values (LQ Suite dPCR) across three lots (lots 1, 2, 3), yielding coefficients of determination R^2^ = 0.9983 (lot 1), 0.9957 (lot 2), and 0.9941 (lot 3) and regression equations y = 1.0239x + 0.0075, y = 1.0020x + 0.0002, and y = 1.0171x + 0.0066, with linear scatter and slopes/intercepts near 1/0 (Fig. 4). Lot‑wise summaries of %IS and MR showed mean %IS of 16.4105%–0.0176% (lot 1), 16.2001%–0.0211% (lot 2), and 15.1063%–0.0159% (lot 3); mean MR of 0.7850–3.8082; %IS bias of −0.7364%–1.2066%; MR bias of −0.0982–0.0815; SD (%IS) of 0.0017%–0.1182%; and %CV of 1.3127%–27.0494% (%IS) and 0.2137%–3.492% (MR), indicating high analytical accuracy and lot‑to‑lot consistency (Supplemental Table 5).

### Clinical validation

**Fig. 5:**
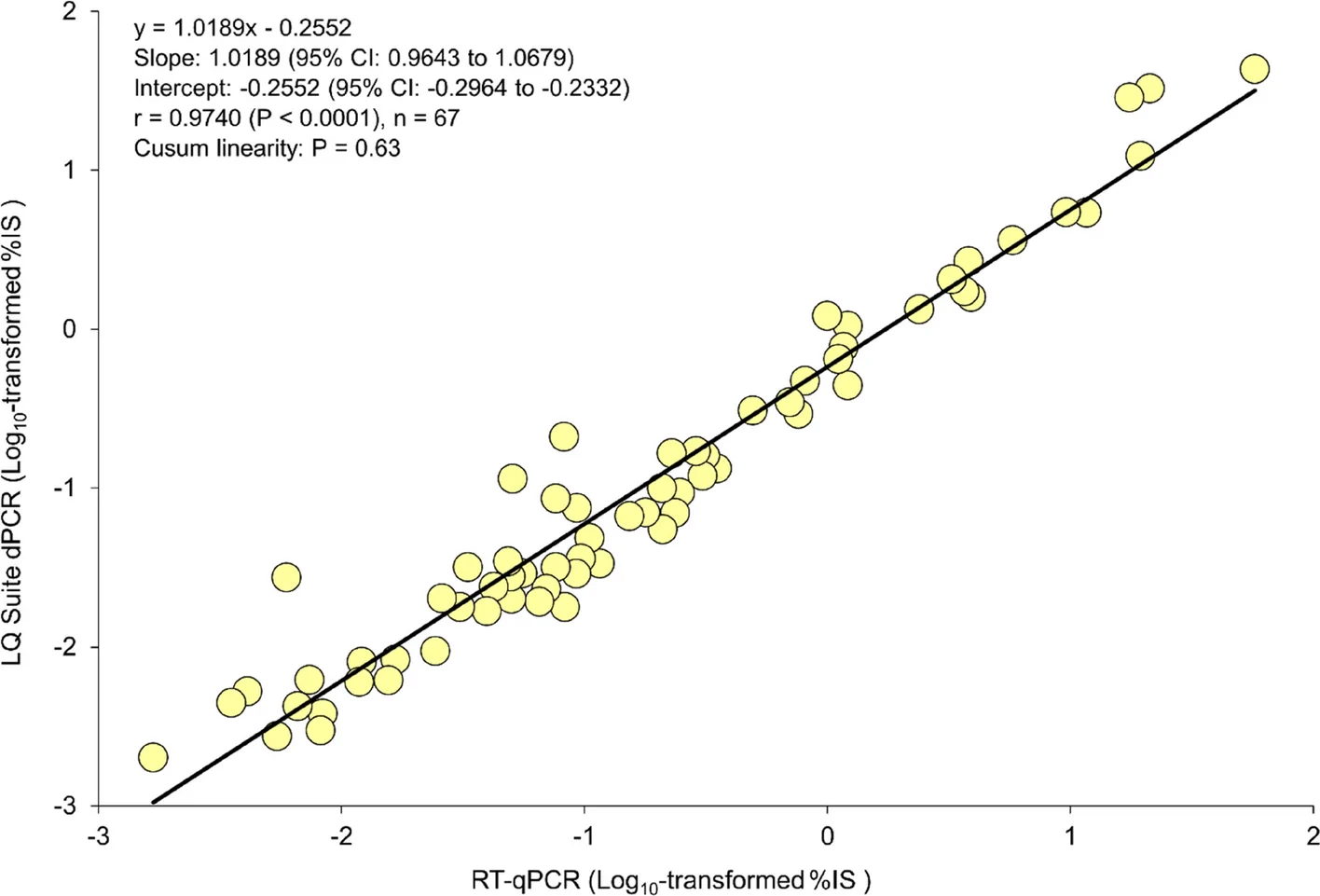
Method comparison between LQ Suite dPCR and RT-qPCR in 67 clinical CML specimens using Passing–Bablok regression analysis. Log_10_-transformed %IS values obtained from LQ Suite dPCR (*y*-axis) were compared with those from RT-qPCR (x-axis). The solid black line represents the regression line. The regression equation is *y* = 1.0189x – 0.2552. The slope of 1.0189 (95% CI: 0.9643 to 1.0679) included 1, indicating no significant proportional bias. Although the intercept of −0.2552 (95% CI: −0.2964 to −0.2332) did not include 0, indicating a minor constant systematic difference, the bias remained well within the predefined acceptable clinical limit (±0.5 log_10_). The Pearson correlation coefficient is r = 0.97 (*P* < 0.0001), demonstrating a strong positive correlation, and the Cusum test confirmed linearity (*P* = 0.63). CI, confidence interval; CML, chronic myeloid leukemia; dPCR, digital PCR; RT-qPCR, real-time quantitative reverse-transcription polymerase chain reaction

**Fig. 6:**
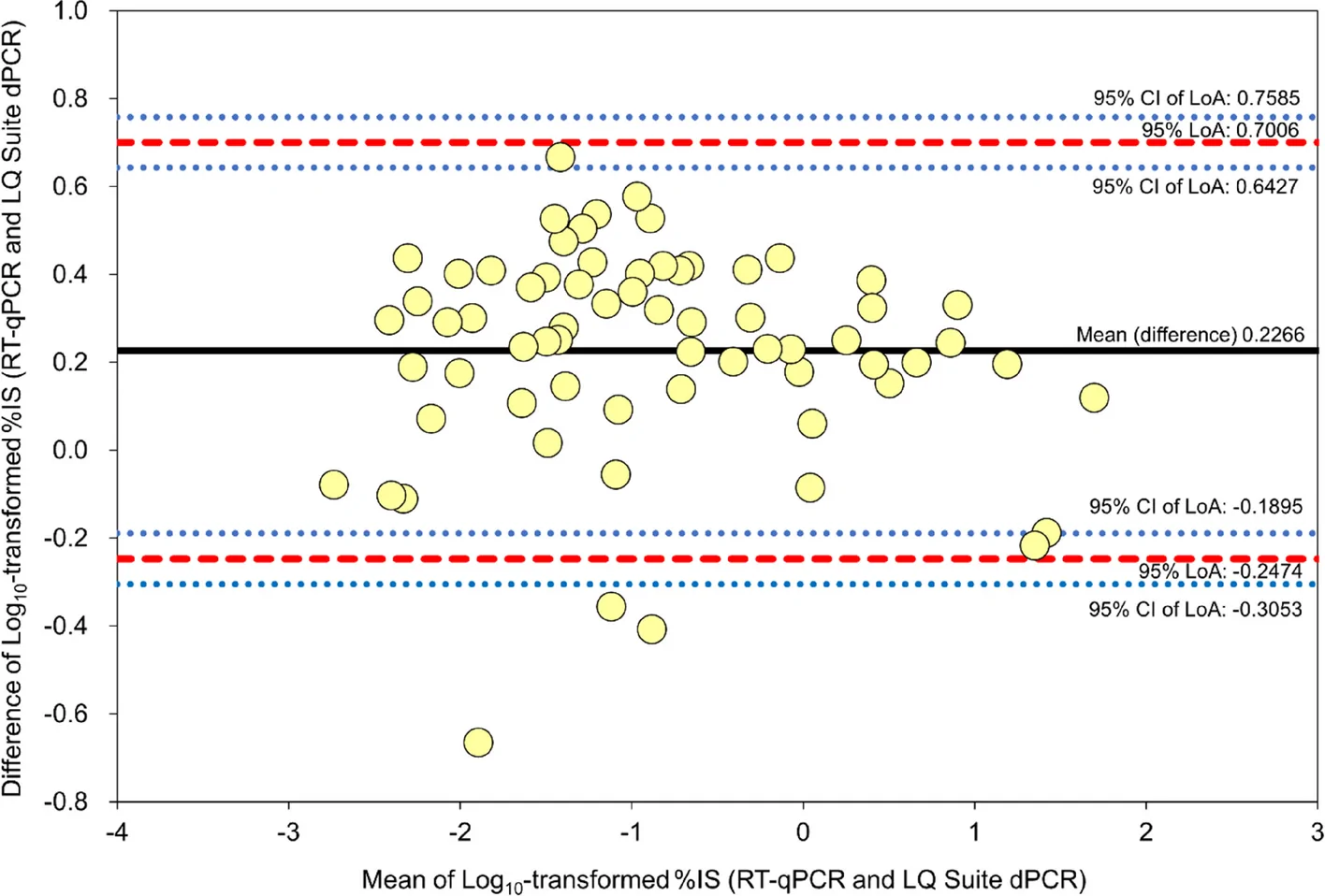
Bland–Altman plot analysis of agreement between RT-qPCR and LQ Suite dPCR for 67 clinical specimens. The *x-*axis represents the mean of Log_10_-transformed %IS values measured by both methods, and *y*-axis represents the difference (RT-qPCR minus LQ Suite dPCR). The solid black line indicates the mean bias of 0.2266, which was within the predefined acceptable clinical limit of ±0.5 log_10_. The red dashed lines represent the 95% limits of agreement, ranging from −0.2474 to 0.7006. The blue dotted lines indicate the 95% CIs for the upper (0.6427 to 0.7585) and lower (−0.3053 to −0.1895) limits of agreement. Most observations fell within the 95% LoA. CI, confidence interval; dPCR, digital PCR; %IS, % International Scale; LoA, limits of agreement; RT-qPCR, real-time quantitative reverse-transcription polymerase chain reaction

**Fig. 7:**
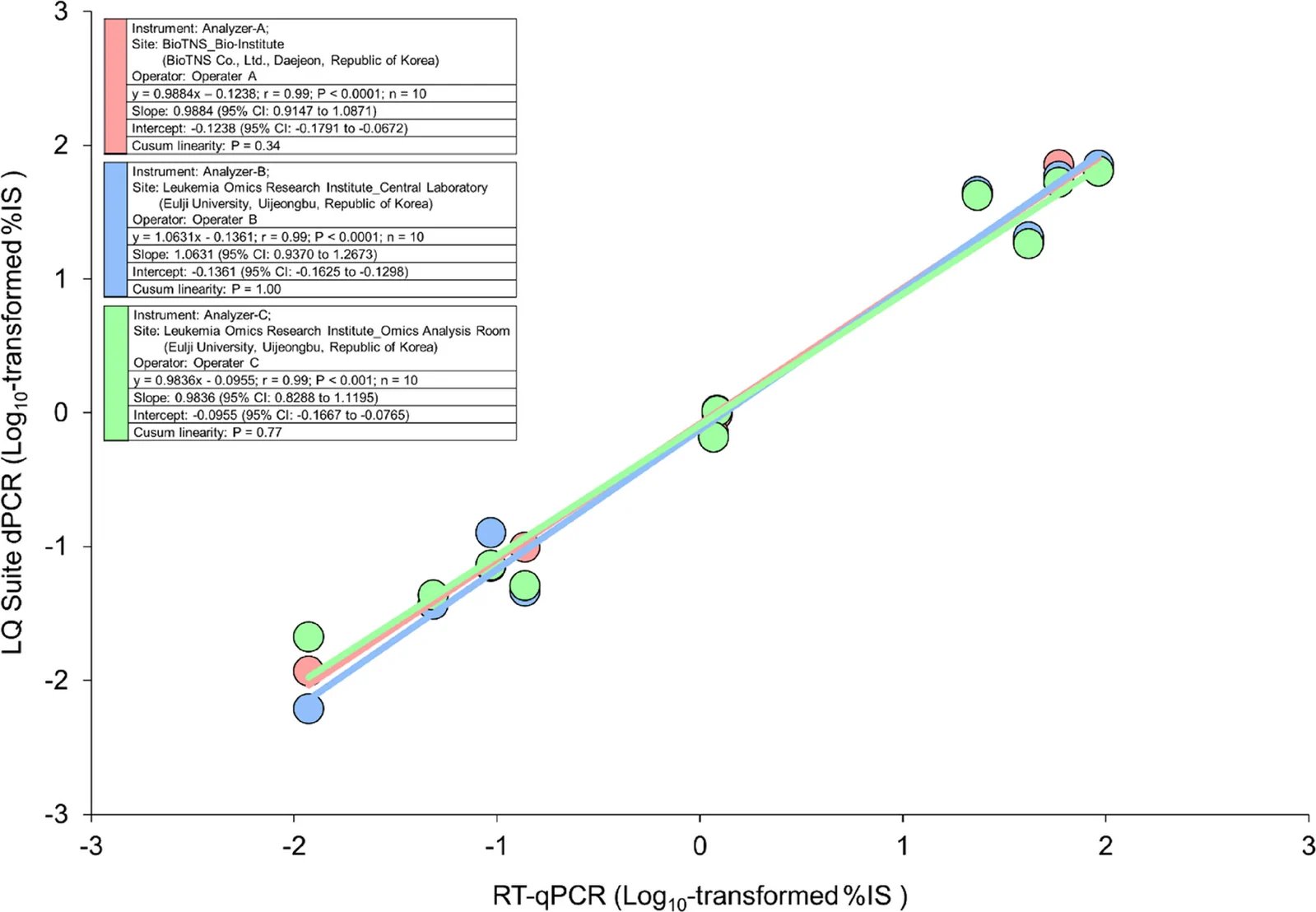
Assessment of inter-laboratory reproducibility using Passing–Bablok regression analysis. Ten clinical CML specimens were analyzed across three different sites, instruments, and operators. The log_10_-transformed %IS values from LQ Suite dPCR (y-axis) were plotted against the RT-qPCR results (x-axis). The regression lines for each condition are color-coded: Red (Site A/Instrument A/Operator A), blue (Site B/Instrument B/Operator B), and green (Site C/Instrument C/Operator C). All three conditions showed excellent correlation (r = 0.99, *P* < 0.0001). The embedded tables detail the regression parameters for each site; in all cases, the 95% CIs for the slopes included 1, confirming the absence of proportional bias. Although the intercepts did not include 0, all constant offsets remained well within the predefined acceptable clinical limit of ±0.5 log_10_, indicating highly reproducible performance across different testing environments. CI, confidence interval; CML, chronic myeloid leukemia; %IS, % International Scale; RT-qPCR, real-time quantitative reverse-transcription polymerase chain reaction

**Fig. 8:**
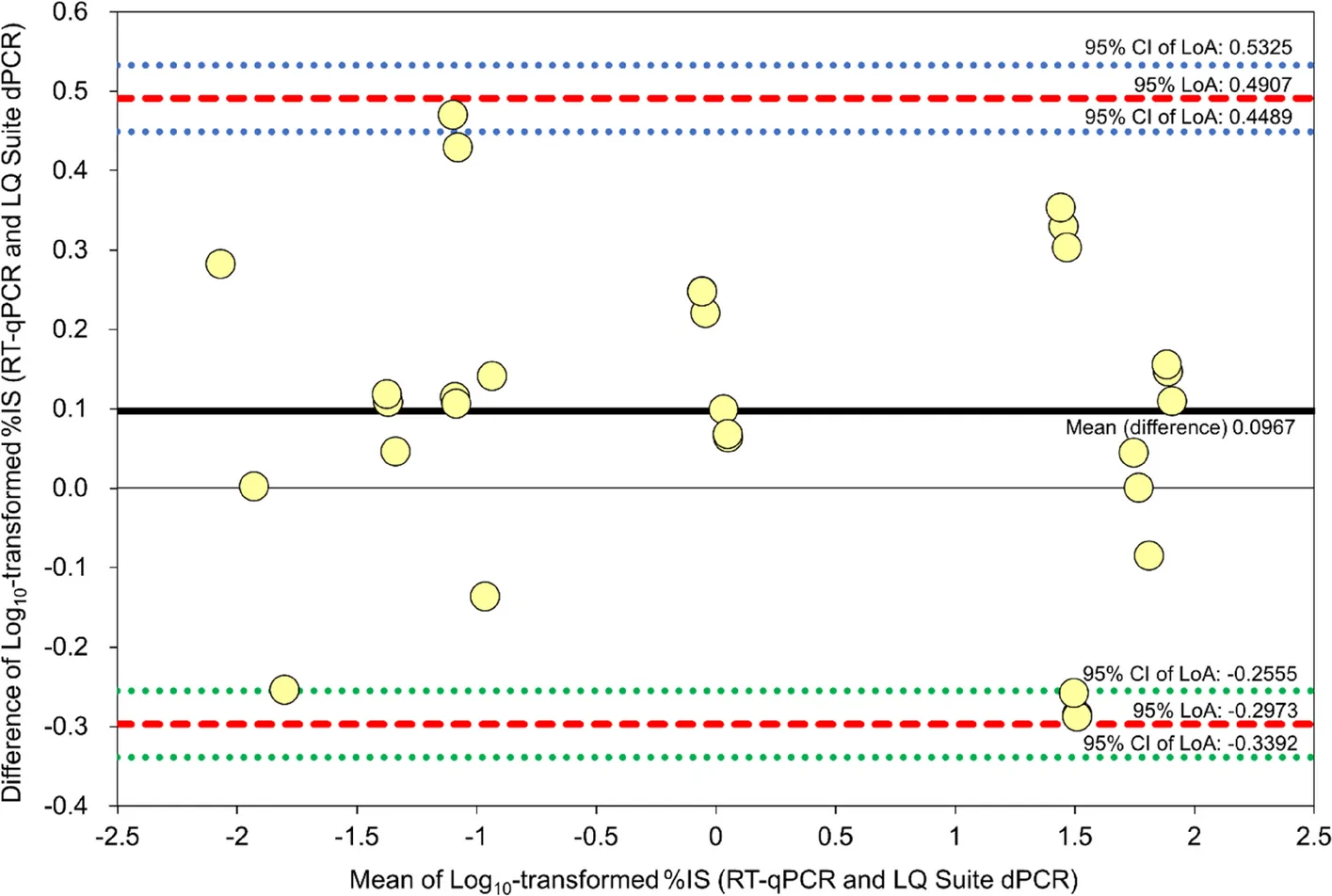
Bland–Altman plot analysis of inter-laboratory reproducibility. The agreement of log_10_-transformed %IS measurements was evaluated across three different sites, instruments, and operators using 10 clinical specimens. The x-axis displays the mean of measurements, and the y-axis displays the difference between measurements. The solid black line represents the mean bias of 0.0967, indicating a negligible systematic difference. The red dashed lines denote the 95% LoA, ranging from −0.2973 to 0.4907. The blue and green dotted lines indicate the 95% confidence intervals (CIs) for the LoA boundaries. All data points fall within the 95% LoA, demonstrating excellent reproducibility and consistency across different testing environments with no outliers. CI, confidence interval; dPCR, digital PCR; %IS, % International Scale; LoA, limits of agreement; RT-qPCR, real-time quantitative reverse-transcription polymerase chain reaction

Clinical performance was evaluated in 72 adult CML specimens from the Republic of Korea for MR-category comparisons between LQ Suite dPCR and RT-qPCR. A quantitative %IS comparison using Passing–Bablok regression and Bland–Altman analysis was performed on 67 specimens with quantitative results from both methods (Figs. 5 and 6). In the 67 quantifiable specimens, Passing–Bablok regression yielded a slope of 1.0189 (95% CI: 0.9643 to 1.0679), which included one, confirming the absence of proportional bias (Fig. 5). The intercept was −0.2552 (95% CI, − 0.2964 to −0.2332); although it did not include zero, this constant systematic difference remained within the predefined acceptable clinical limit of ±0.5 log_10_ (Fig. 5). For the log_10_-transformed %IS, the methods were strongly correlated (r = 0.9740, *P* < 0.0001) (Fig. 5). Bland–Altman analysis indicated a mean bias of 0.2266 (RT-qPCR minus LQ Suite dPCR), with most observations falling within the 95% limit of agreement (Fig. 6). MR-category agreement in the 72-specimen categorical comparison was moderate (Cohen’s kappa = 0.54), with 63.9% (46/72) concordance. Among the 26 discordant cases, 20 were classified as having deeper MR by dPCR, one was classified as having deeper MR by RT-qPCR, and 5 showed low-level positivity by RT-qPCR but negative dPCR results, according to the predefined LoB-based reporting criterion (Table 7). Reproducibility across three sites, instruments, and operators using 10 specimens spanning MR 0.04–3.93 met the criteria at each site, with slope 95% CIs including 1, constant offsets within the predefined ±0.5 log_10_ clinical limit, and r = 0.99 correlation (Fig. 7). Bland–Altman analysis of log_10_-transformed %IS demonstrated a low mean bias of 0.0967, with all observations within the 95% limit of agreement and no outliers (Fig. 8).

**Table 7:** Comparison of MR category classifications between LQ Suite dPCR and RT-qPCR

			LQ Suite dPCR System (n)					
	MR level	> MR1	MR2	MR3	MR4	MR4.5	Negative	Sum
RT-qPCR (n)	> MR1	**7**	4^a^	-	-	-	-	11
	MR2	-	**8**	3^a^		-	-	11
	MR3	-	-	**20**	9^a^		1^c^	30
	MR4	-	-	-	**2**	4^a^	4^c^	10
	MR4.5	-	-	-	1^b^	9	-	10
	Negative	-	-	-	-	-	-	0
	Sum	7	12	23	12	13	5	72

Bold font indicates concordant classifications

ᵃCases in which dPCR classified specimens as having a deeper molecular response than RT-qPCR. This pattern may reflect enhanced analytical sensitivity of dPCR. However, stochastic variation at low target concentrations or pre-analytical variation in retrospectively archived total RNA may also contribute to shifts between adjacent MR categories

ᵇCase in which RT-qPCR classified a specimen as having a deeper molecular response than dPCR, possibly due to stochastic variation near the low-copy detection limit

^c^Cases with low-level positivity by RT-qPCR (MR3.92–4.47) but negative dPCR results according to the predefined reporting criterion (≤ 1 positive droplet, below the limit of blank). These valid categorical negative results were included in the MR-category agreement analysis but excluded from the paired log-transformed quantitative analyses. The moderate agreement (κ = 0.54) reflects discordance in MR-category classification between the methods. Clinical significance of these discrepancies requires longitudinal validation

*dPCR* digital PCR, *gDNA* genomic DNA, *MR* molecular response, *RT-qPCR* real-time quantitative reverse-transcription polymerase chain reaction, *SD* standard deviation

## Discussion

This study validated the analytical and clinical performance of the LQ Check BCR::ABL1 Major %IS Kit, demonstrating high sensitivity, precision, specificity, accuracy, and reproducibility compared with conventional RT‑qPCR. The LoD and LoQ were established at 0.0025%IS (MR4.6), exceeding the sensitivity required to detect the MR4.5 (0.0032%IS) threshold recommended by major guidelines for considering TFR [28]. This finding contrasts with previous reports that dPCR sensitivity may be limited to MR4.0 (0.01%IS) owing to false positives [29]. The LoQ of MR4.6 was established using a study-specific criterion of CV ≤ 76%, adopted from a precedent involving an FDA-cleared dPCR assay [17]. Because this is not a universally recommended CLSI cutoff, the resulting LoQ should not be generalized to other *BCR::ABL1* dPCR assays.

A quantitative comparison involving 67 Korean CML specimens showed a high correlation (r = 0.9740) and a Bland–Altman mean difference of 0.2266 (RT-qPCR minus LQ Suite dPCR). In the categorical comparison involving 72 specimens, 20 were assigned to deeper MR categories by dPCR, whereas five specimens with low-level positive RT-qPCR results were negative by dPCR according to the predefined LoB-based reporting criterion. Although differences in analytical sensitivity may contribute to these discordances, stochastic variation in target molecule sampling at very low concentrations, followed by Poisson-based partitioning, may also lead to changes between the assays [30]. Furthermore, differences in template integrity or freeze–thaw effects associated with retrospectively archived RNA specimens may have influenced agreement between the assays. Without longitudinal follow-up after TKI discontinuation, whether these deeper MR classifications are associated with improved TFR outcomes remains unknown.

Reproducibility was high across the three institutions, instruments, and operators, supporting the potential for multicenter standardization (Figs. 7 and 8). Analytical specificity testing demonstrated no interference from endogenous or exogenous substances and no cross-reactivity with related genes (Tables 5 and 6). Accuracy testing also confirmed WHO traceability, enabling laboratories to report results directly on the %IS using lot-specific conversion factors (Fig. 4). The clinical relevance of material‑dependent bias also warrants consideration. Passing–Bablok regression of results from 67 clinical specimens yielded a slope of 1.0189 (95% CI, 0.9643 to 1.0679), indicating that the proportional bias observed in cell‑line dilutions (slope, 0.87–0.90) was largely absent in clinical specimens, with the slope approaching the ideal value of 1. This finding supports the assay’s robustness in patient samples (Fig. 5). Although the intercept of −0.2552 (95% CI, − 0.2964 to −0.2332) did not include 0, indicating a small constant systematic difference, this offset remained well within the predefined clinically acceptable equivalence margin of ±0.5 Log_10_, supporting analytical and clinical comparability between the assays.

Compared with existing droplet-based platforms, the LQ Suite dPCR system offers a streamlined, cost-effective workflow that may reduce hands-on time in clinical laboratories. Regarding specimen handling, total RNA extraction within 48 h is preferred; 72 h represents an exceptional upper limit, with reporting contingent on *BCR* ≥ 2,000 copies/μL.

Although the study was limited by its pilot scale (n = 72) and the retrospective nature of the archived specimen cohort, strict adherence to multiple CLSI guidelines supports the analytical reliability of the assay for clinical implementation. Future multicenter international studies incorporating longitudinal clinical outcomes are necessary to establish the generalizability of these findings and determine clinical implications for TFR, particularly for the major transcripts that account for > 90% of patients with CML.

In conclusion, the LQ Suite dPCR system provides reliable, reproducible *BCR::ABL1* quantification at MR4.6 within the clinically critical DMR range, supporting standardized MRD reporting. However, the observed tendency toward deeper MR classification requires confirmation in prospective studies evaluating TFR outcomes to establish its clinical significance.

## Supplementary Information

Supplementary Material 1.

## Data Availability

All data supporting the findings of this study are available within the paper and its Supplementary Information.
