# Supplementary material for: Next-generation digital polymerase chain reaction assay for BCR::ABL1 in chronic myeloid leukemia: comprehensive validation for standardized measurable residual disease detection
Source: Blood Res. 2026 Oct 16;61(1):67. doi: 10.1007/s44313-026-00188-5 (PMC13675441; doi:10.1007/s44313-026-00188-5)
Supplement: Supplementary file 1 [file 44313_2026_188_MOESM1_ESM.docx]

**RESEARCH ARTICLE**

**Next-Generation Digital Polymerase Chain Reaction Assay for *BCR::ABL1* in Chronic Myeloid Leukemia: Comprehensive Validation Enabling Standardized Measurable Residual Disease Detection**

**Yong-Hwi Kim^1^, Min-Sik Jang^1^, Shin-Hyun Kim^1^, In-Young Roh^1^, Bong-Suk Kim^1^, Kyung-Mi Kee^2^, Soo-Hyun Kim^2^, Dong-Wook Kim^3^, and Jae-Hoon Kim^1*^**

**^1^BioTNS Co., Ltd., Daejeon 34183, Republic of Korea**

**^2^Leukemia Omics Research Institute, Eulji University, Uijeongbu 11759, Republic of Korea**

**^3^Department of Hematology, Hematology Center, Eulji Medical Center, Eulji University, Uijeongbu 11759, Republic of Korea**

**^*^** Correspondence to: Jae-Hoon Kim, Ph.D.

BioTNS Co., Ltd., Daejeon 34183, Republic of Korea

E-mail: jhkim@biotns.co.kr
Supplementary Table 1. Limit of blank (LoB) analysis results based on 60 replicate measurements using eight HL‑60 negative cell line samples (*BCR::ABL1* fusion–negative)

| **No.** | **Total droplet** | ***BCR::ABL1*^1^** | | ***BCR*^2^** | |  | **No.** | **Total droplet** | ***BCR::ABL1*** | | ***BCR*** | |
| --- | --- | --- | --- | --- | --- | --- | --- | --- | --- | --- | --- | --- |
|  |  | **Positive droplet** | **Conc.^3^** | **Positive droplet** | **Conc.** |  |  |  | **Positive droplet** | **Conc.*** | **Positive droplet** | **Conc.** |
| 1 | 20,084 | 0.00 | 0.00 | 19,252 | 8,019.76 |  | 31 | 21,445 | 0 | 0.00 | 19,972 | 6,746.07 |
| 2 | 18,596 | 0.00 | 0.00 | 17,519 | 7,175.74 |  | 32 | 22,385 | 0 | 0.00 | 21,334 | 7,704.41 |
| 3 | 19,493 | 0.00 | 0.00 | 18,448 | 7,370.37 |  | 33 | 22,099 | 0 | 0.00 | 20,656 | 6,873.57 |
| 4 | 17,943 | 0.00 | 0.00 | 17,123 | 7,772.42 |  | 34 | 22,104 | 0 | 0.00 | 20,003 | 5,927.82 |
| 5 | 19,539 | 0.00 | 0.00 | 18,591 | 7,621.70 |  | 35 | 18,217 | 0 | 0.00 | 17,305 | 7,542.75 |
| 6 | 19,152 | 0.00 | 0.00 | 18,440 | 8,292.40 |  | 36 | 19,345 | 0 | 0.00 | 18,133 | 6,977.74 |
| 7 | 20,377 | 0.00 | 0.00 | 19,481 | 7,869.58 |  | 37 | 17,842 | 0 | 0.00 | 16,884 | 7,366.40 |
| 8 | 19,625 | 0.00 | 0.00 | 18,424 | 7,036.90 |  | 38 | 19,559 | 0 | 0.00 | 18,391 | 7,098.60 |
| 9 | 17,870 | 0.00 | 0.00 | 17,432 | 9,341.71 |  | 39 | 19,051 | 0 | 0.00 | 17,514 | 6,340.77 |
| 10 | 19,407 | 0.00 | 0.00 | 18,590 | 7,979.22 |  | 40 | 24,214 | 0 | 0.00 | 23,113 | 7,785.17 |
| 11 | 16,612 | 0.00 | 0.00 | 15,806 | 7,621.65 |  | 41 | 23,749 | 0 | 0.00 | 21,218 | 5,639.61 |
| 12 | 19,021 | 0.00 | 0.00 | 18,217 | 7,969.02 |  | 42 | 19,997 | 0 | 0.00 | 18,885 | 7,278.14 |
| 13 | 22,795 | 0.00 | 0.00 | 21,411 | 7,056.83 |  | 43 | 24,271 | 0 | 0.00 | 22,885 | 7,211.24 |
| 14 | 19,101 | 0.00 | 0.00 | 18,455 | 8,530.72 |  | 44 | 19,552 | 0 | 0.00 | 18,736 | 8,001.05 |
| 15 | 17,697 | 0.00 | 0.00 | 17,170 | 8,851.26 |  | 45 | 21,944 | 0 | 0.00 | 21,229 | 8,624.60 |
| 16 | 17,719 | 0.00 | 0.00 | 16,943 | 7,879.70 |  | 46 | 16,053 | 0 | 0.00 | 15,347 | 7,869.11 |
| 17 | 19,338 | 0.00 | 0.00 | 18,329 | 7,438.57 |  | 47 | 14,992 | 0 | 0.00 | 13,858 | 6,503.19 |
| 18 | 19,068 | 0.00 | 0.00 | 18,636 | 9,539.90 |  | 48 | 24,959 | 0 | 0.00 | 22,313 | 5,652.86 |
| 19 | 20,467 | 0.00 | 0.00 | 19,449 | 7,559.13 |  | 49 | 18,627 | 0 | 0.00 | 17,542 | 7,161.29 |
| 20 | 19,649 | 0.00 | 0.00 | 18,632 | 7,458.86 |  | 50 | 17,974 | 0 | 0.00 | 17,294 | 8,248.33 |
| 21 | 18,471 | 0.00 | 0.00 | 17,686 | 7,955.35 |  | 51 | 18,675 | 0 | 0.00 | 16,908 | 5,939.30 |
| 22 | 23,572 | 0.00 | 0.00 | 20,994 | 5,574.42 |  | 52 | 18,752 | 0 | 0.00 | 17,742 | 7,358.56 |
| 23 | 24,383 | 0.00 | 0.00 | 23,332 | 7,919.76 |  | 53 | 23,132 | 1 | 0.11 | 21,667 | 6,950.53 |
| 24 | 20,422 | 0.00 | 0.00 | 19,325 | 7,365.32 |  | 54 | 20,896 | 1 | 0.12 | 19,865 | 7,579.42 |
| 25 | 17,321 | 0.00 | 0.00 | 16,191 | 6,875.82 |  | 55 | 19,034 | 1 | 0.13 | 18,055 | 7,474.69 |
| 26 | 21,893 | 0.00 | 0.00 | 20,058 | 6,244.64 |  | 56 | 18,201 | 1 | 0.14 | 16,145 | 5,492.98 |
| 27 | 21,491 | 0.00 | 0.00 | 19,167 | 5,602.88 |  | 57 | 17,853 | 1 | 0.14 | 16,549 | 6,591.27 |
| 28 | 19,301 | 0.00 | 0.00 | 18,416 | 7,764.04 |  | 58 | 16,202 | 1 | 0.16 | 15,656 | 8,539.73 |
| 29 | 24,543 | 0.00 | 0.00 | 22,920 | 6,841.69 |  | 59 | 16,096 | 1 | 0.16 | 15,124 | 7,070.45 |
| 30 | 18,593 | 0.00 | 0.00 | 17,498 | 7,133.58 |  | 60 | 15,589 | 1 | 0.16 | 14,680 | 7,158.63 |

^1^, FAM fluorescence; ^2^, HEX fluorescence; ^3^, copies/μL.

Supplementary Table 2. Limit of detection (LoD) and Limit of quantitation (LoQ) results for each *BCR::ABL1* transcript type (e13a2, e14a2) based on cell line–derived serial dilution panels (K‑562 and KCL‑22 spiked into HL‑60), determined using a non-parametric approach

| Lot | Transcript identity | Total specimens | Positive specimens | Positive detection rate (%) | CV (%) | | Molecular indicator | | Results |
| --- | --- | --- | --- | --- | --- | --- | --- | --- | --- |
|  |  |  |  |  | %IS | MR | %IS | MR |  |
| 1 | e13a2 | 24 | 24 | 100.00 | 34.35 | 3.54 | 0.0032 | 4.49 | Pass |
| 1 | e14a2 | 24 | 24 | 100.00 | 48.83 | 4.42 | 0.0032 | 4.49 | Pass |
| **1** | **e13a2** | **24** | **23** | **95.83** | **42.21** | **5.13** | **0.0025** | **4.60** | **Pass** |
| **1** | **e14a2** | **24** | **23** | **95.83** | **37.83** | **4.17** | **0.0025** | **4.60** | **Pass** |
| 1 | e13a2 | 24 | 17 | 70.83 | 68.47 | 5.55 | 0.0020 | 4.70 | Fail |
| 1 | e14a2 | 24 | 17 | 70.83 | 72.83 | 4.36 | 0.0020 | 4.70 | Fail |
| 1 | e13a2 | 24 | 10 | 41.67 | 93.46 | 6.89 | 0.0016 | 4.80 | Fail |
| 1 | e14a2 | 24 | 13 | 54.17 | 98.70 | 5.46 | 0.0016 | 4.80 | Fail |
| 1 | e13a2 | 24 | 10 | 41.67 | 107.98 | 7.99 | 0.0013 | 4.89 | Fail |
| 1 | e14a2 | 24 | 10 | 41.67 | 111.98 | 6.16 | 0.0013 | 4.89 | Fail |
| 1 | e13a2 | 24 | 8 | 33.33 | 122.37 | 7.31 | 0.0010 | 5.00 | Fail |
| 1 | e14a2 | 24 | 7 | 29.17 | 159.74 | 5.38 | 0.0010 | 5.00 | Fail |
| 2 | e13a2 | 24 | 24 | 100.00 | 39.03 | 4.21 | 0.0032 | 4.49 | Pass |
| 2 | e14a2 | 24 | 24 | 100.00 | 36.14 | 4.06 | 0.0032 | 4.49 | Pass |
| **2** | **e13a2** | **24** | **23** | **95.83** | **41.60** | **4.37** | **0.0025** | **4.60** | **Pass** |
| **2** | **e14a2** | **24** | **23** | **95.83** | **52.06** | **4.30** | **0.0025** | **4.60** | **Pass** |
| 2 | e13a2 | 24 | 18 | 75.00 | 79.65 | 7.69 | 0.0020 | 4.70 | Fail |
| 2 | e14a2 | 24 | 17 | 70.83 | 75.81 | 5.27 | 0.0020 | 4.70 | Fail |
| 2 | e13a2 | 24 | 10 | 41.67 | 124.49 | 6.60 | 0.0016 | 4.80 | Fail |
| 2 | e14a2 | 24 | 14 | 58.33 | 82.79 | 7.49 | 0.0016 | 4.80 | Fail |
| 2 | e13a2 | 24 | 9 | 37.50 | 127.51 | 9.18 | 0.0013 | 4.89 | Fail |
| 2 | e14a2 | 24 | 9 | 37.50 | 131.76 | 9.55 | 0.0013 | 4.89 | Fail |
| 2 | e13a2 | 24 | 11 | 45.83 | 111.84 | 6.76 | 0.0010 | 5.00 | Fail |
| 2 | e14a2 | 24 | 10 | 41.67 | 116.57 | 7.92 | 0.0010 | 5.00 | Fail |

CV, coefficient of variation; %IS, % international scale; MR, molecular response. Bold indicates the LoD and LoQ ranges.

Supplementary Table 3. Linearity assessment for the major *BCR::ABL1* fusion transcript (e13a2)

| Lot 1 | | | | |  | Lot 2 | | | | |
| --- | --- | --- | --- | --- | --- | --- | --- | --- | --- | --- |
| Samples | %IS | Mean | MR | Mean |  | Samples | %IS | Mean | MR | Mean |
| 10.4301 %IS (MR0.9817) | 8.6624 | 8.2355 | 1.0624 | 1.0854 |  | 10.4301 %IS  (MR0.9817) | 8.6068 | 8.8236 | 1.0652 | 1.0561 |
|  | 7.4337 |  | 1.1288 |  |  |  | 9.8999 |  | 1.0044 |  |
|  | 8.6103 |  | 1.0650 |  |  |  | 7.9640 |  | 1.0989 |  |
| 1.1549 %IS  (MR1.9375) | 1.0332 | 1.1629 | 1.9858 | 1.9380 |  | 1.1549 %IS  (MR1.9375) | 1.1323 | 1.1468 | 1.9460 | 1.9438 |
|  | 1.3765 |  | 1.8612 |  |  |  | 1.3266 |  | 1.8772 |  |
|  | 1.0791 |  | 1.9669 |  |  |  | 0.9813 |  | 2.0082 |  |
| 0.1282 %IS  (MR2.8921) | 0.0979 | 0.0968 | 3.0092 | 3.0143 |  | 0.1282 %IS  (MR2.8921) | 0.1485 | 0.1166 | 2.8284 | 2.9414 |
|  | 0.0915 |  | 3.0388 |  |  |  | 0.0941 |  | 3.0265 |  |
|  | 0.1012 |  | 2.9950 |  |  |  | 0.1073 |  | 2.9692 |  |
| 0.0101 %IS  (MR3.9966) | 0.0129 | 0.0134 | 3.8908 | 3.8799 |  | 0.0101 %IS  (MR3.9966) | 0.0176 | 0.0168 | 3.7557 | 3.7752 |
|  | 0.0109 |  | 3.9619 |  |  |  | 0.0163 |  | 3.7888 |  |
|  | 0.0163 |  | 3.7869 |  |  |  | 0.0166 |  | 3.7812 |  |
| 0.0023 %IS  (MR4.6399) | 0.0042 | 0.0047 | 4.3782 | 4.3313 |  | 0.0023 %IS  (MR4.6399) | 0.0051 | 0.0055 | 4.2944 | 4.2638 |
|  | 0.0048 |  | 4.3224 |  |  |  | 0.0060 |  | 4.2214 |  |
|  | 0.0051 |  | 4.2933 |  |  |  | 0.0053 |  | 4.2755 |  |
| 0.0006 %IS  (MR5.2596) | 0.0022 | 0.0024 | 4.6557 | 4.6277 |  | 0.0006 %IS  (MR5.2596) | 0.0018 | 0.0039 | 4.7546 | 4.4569 |
|  | 0.0028 |  | 4.5577 |  |  |  | 0.0060 |  | 4.2236 |  |
|  | 0.0021 |  | 4.6697 |  |  |  | 0.0040 |  | 4.3926 |  |
| Linearity (R^2^) | 0.9928 | | | |  | Linearity (R^2^) | 0.9939 | | | |
| CF | 0.8001 | | | |  | CF | 1.0836 | | | |

*, Negative. Evaluated with two independent lots (not the same as analytical accuracy lots).

Supplementary Table 4. Linearity assessment for the major *BCR::ABL1* fusion transcript (e14a2)

| Lot 1 | | | | |  | Lot 2 | | | | |
| --- | --- | --- | --- | --- | --- | --- | --- | --- | --- | --- |
| Samples | %IS | Mean | MR | Mean |  | Samples | %IS | Mean | MR | Mean |
| 10.6813 %IS  (MR0.9714) | 10.4742 | 10.4713 | 0.9799 | 0.9800 |  | 10.6813 %IS  (MR0.9714) | 8.8772 | 9.3911 | 1.0517 | 1.0280 |
|  | 10.4792 |  | 0.9797 |  |  |  | 9.1499 |  | 1.0386 |  |
|  | 10.4606 |  | 0.9804 |  |  |  | 10.1463 |  | 0.9937 |  |
| 1.0500 %IS  (MR1.9788) | 0.8242 | 0.8347 | 2.0840 | 2.0795 |  | 1.0500 %IS  (MR1.9788) | 1.0390 | 1.0164 | 1.9834 | 1.9950 |
|  | 0.7689 |  | 2.1141 |  |  |  | 0.8866 |  | 2.0523 |  |
|  | 0.9110 |  | 2.0405 |  |  |  | 1.1235 |  | 1.9494 |  |
| 0.0990 %IS  (MR3.0045) | 0.1356 | 0.1242 | 2.8679 | 2.9113 |  | 0.0990 %IS  (MR3.0045) | 0.1262 | 0.1299 | 2.8988 | 2.8899 |
|  | 0.0977 |  | 3.0103 |  |  |  | 0.1117 |  | 2.9521 |  |
|  | 0.1394 |  | 2.8557 |  |  |  | 0.1517 |  | 2.8190 |  |
| 0.0082 %IS  (MR4.0838) | 0.0119 | 0.0112 | 3.9232 | 3.9530 |  | 0.0082 %IS  (MR4.0838) | 0.0136 | 0.0143 | 3.8675 | 3.8459 |
|  | 0.0093 |  | 4.0333 |  |  |  | 0.0156 |  | 3.8057 |  |
|  | 0.0125 |  | 3.9026 |  |  |  | 0.0137 |  | 3.8644 |  |
| 0.0022 %IS  (MR4.6484) | 0.0059 | 0.0056 | 4.2294 | 4.2670 |  | 0.0022 %IS  (MR4.6484) | 0.0064 | 0.0060 | 4.1966 | 4.2187 |
|  | 0.0037 |  | 4.4299 |  |  |  | 0.0059 |  | 4.2325 |  |
|  | 0.0072 |  | 4.1417 |  |  |  | 0.0059 |  | 4.2271 |  |
| 0.0010 %IS  (MR4.9792) | 0.0007 | 0.0016 | 5.1493 | 4.8475 |  | 0.0010 %IS  (MR4.9792) | 0.0052 | 0.0054 | 4.2841 | 4.2768 |
|  | 0.0022 |  | 4.6495 |  |  |  | 0.0064 |  | 4.1911 |  |
|  | 0.0018 |  | 4.7438 |  |  |  | 0.0044 |  | 4.3552 |  |
| Linearity (R^2^) | 0.9916 | | | |  | Linearity (R^2^) | 0.9972 | | | |
| CF | 0.8001 | | | |  | CF | 1.0836 | | | |

*, Negative. Evaluated with two independent lots (not the same as analytical accuracy lots).

Supplementary Table 5. Comparison of %IS and MR values across three lots of the LQ Check BCR::ABL1 Major %IS Kit, traceable to the WHO primary reference standard

| Lot 1 | | | | Lot 2 | | | | Lot 3 | | | |
| --- | --- | --- | --- | --- | --- | --- | --- | --- | --- | --- | --- |
| Samples | | %IS | MR | Samples | | %IS | MR | Samples | | %IS | MR |
| 08/198  (16.3129 %IS,  MR0.7875) | Mean | 16.4105 | 0.7850 | 08/198  (16.3129 %IS,  MR0.7875) | Mean | 16.2001 | 0.7911 | 08/198  (16.3129 %IS,  MR0.7875) | Mean | 15.1063 | 0.8209 |
|  | Bias | -0.0976 | 0.0025 |  | Bias | -0.1128 | -0.0036 |  | Bias | 1.2066 | -0.0335 |
|  | SD | 0.4566 | 0.0121 |  | SD | 1.0470 | 0.0276 |  | SD | 0.3510 | 0.0100 |
|  | CV (%) | 2.7827 | 1.5432 |  | CV (%) | 6.4632 | 3.4920 |  | CV (%) | 2.3233 | 1.2229 |
| 08/196  (1.6627 %IS,  MR1.7792) | Mean | 1.9564 | 1.7096 | 08/196  (1.6627 %IS,  MR1.7792) | Mean | 1.8240 | 1.7407 | 08/196  (1.6627 %IS,  MR1.7792) | Mean | 1.9066 | 1.7202 |
|  | Bias | -0.2937 | 0.0696 |  | Bias | -0.1613 | 0.0385 |  | Bias | -0.2439 | 0.0590 |
|  | SD | 0.1694 | 0.0379 |  | SD | 0.1937 | 0.0477 |  | SD | 0.1071 | 0.0247 |
|  | CV (%) | 8.6563 | 2.2148 |  | CV (%) | 10.6183 | 2.7412 |  | CV (%) | 5.6169 | 1.4378 |
| 08/194  (0.1753 %IS,  MR2.7562) | Mean | 0.1675 | 2.7776 | 08/194  (0.1753 %IS,  MR2.7562) | Mean | 0.1545 | 2.8152 | 08/194  (0.1753 %IS,  MR2.7562) | Mean | 0.2115 | 2.6747 |
|  | Bias | 0.0078 | -0.0213 |  | Bias | 0.0208 | -0.0590 |  | Bias | -0.0362 | 0.0815 |
|  | SD | 0.0177 | 0.0468 |  | SD | 0.0250 | 0.0735 |  | SD | 0.0028 | 0.0057 |
|  | CV (%) | 10.5500 | 1.6866 |  | CV (%) | 16.2156 | 2.6107 |  | CV (%) | 1.3127 | 0.2137 |
| 08/192  (0.0195 %IS,  MR3.7100) | Mean | 0.0176 | 3.7552 | 08/192  (0.0195 %IS,  MR3.7100) | Mean | 0.0211 | 3.6873 | 08/192  (0.0195 %IS,  MR3.7100) | Mean | 0.0159 | 3.8082 |
|  | Bias | 0.0019 | -0.0453 |  | Bias | -0.0016 | 0.0227 |  | Bias | 0.0036 | -0.0982 |
|  | SD | 0.0017 | 0.0429 |  | SD | 0.0057 | 0.1182 |  | SD | 0.0043 | 0.1160 |
|  | CV (%) | 9.6825 | 1.1434 |  | CV (%) | 27.0494 | 3.2044 |  | CV (%) | 26.7040 | 3.0460 |
| CF | 1.28 | | | CF | 1.06 | | | CF | 1.07 | | |

Evaluated with three independent lots (different from linearity lots).
